# Bacterial communities associated with solid waste leachates (SWL) at Olusosun and Oke-Afa dumpsites in Lagos State, Nigeria

**DOI:** 10.1128/mra.00085-25

**Published:** 2025-08-05

**Authors:** Adewale K. Ogunyemi, Olanike M. Buraimoh, Bukola C. Ogunyemi, Emmanuel O. Olumuyiwa, Samuel A. Osunnaya, Simeon K. Odetunde, Olukemi A. Tobun, Titilola A. Samuel, Matthew O. Ilori, Olukayode O. Amund

**Affiliations:** 1 Department of Biological Sciences (Microbiology Unit), Trinity University, Yaba, Lagos State, Nigeria; 2 Department of Microbiology, University of Lagos 70670, Akoka, Lagos State, Nigeria; 3 TETFund Centre of Excellence on Biodiversity Conservation and Ecosystem Management (TCEBCEM), University of Lagos 70670, Yaba, Lagos State, Nigeria; 4 Department of Biochemistry, University of Lagos 70670, Id-Araba, Lagos State, Nigeria; 5 Department of Biological Sciences, Bells University of Technology 300183, Ota, Ogun State, Nigeria; 6 Department of Molecular Biology and Biotechnology, Nigerian Institute of Medical Research 206896, Yaba, Lagos State, Nigeria; 7 Department of Biological Sciences, Lagos State University of Science and Technology 383260, Ikorodu, Lagos State, Nigeria; DOE Joint Genome Institute, Berkeley, California, USA

**Keywords:** 16S rRNA gene sequencing, bacterial communities, solid waste leachates (SWL), dumpsites, firmicutes

## Abstract

Here, we use 16S rRNA gene sequencing to investigate bacterial community profiles at Olusosun and Oke-Afa dumpsites. The Olusosun and Oke-Afa dumpsites had 305 and 306 operational taxonomic units (OTUs) detected, respectively, while the Olusosun control site had 233 OTUs detected. *Firmicutes* are the major phylum in all samples. This study reveals highly diverse bacteria communities at dumpsites with potential for bioprospecting for better waste control.

## ANNOUNCEMENT

Solid waste represents a significant global environmental issue today, affecting both less developed and developed countries.

In recent decades, the rise in population, economic growth, and community living standards has all contributed to an increase in solid waste generation (1, 2). Afolagboye et al. (3) report that solid waste is being generated in parallel with a rapid increase in population and urbanization. Invariably, changes in the chemical composition of waste disposed contribute to their association with a diverse group of microorganisms (4, 5). The present study assessed the bacterial community structure in solid waste leachates at Olusosun and Oke-Afa dumpsites, Lagos State, Nigeria. Olusosun dumpsite, located in the Ojota region, is the most active dumpsite in Lagos State (6), Sub-Saharan Africa’s most populous city. The second dumpsite used in this present study is Oke-Afa, located in the Oshodi/Isolo region of Lagos State.

The solid waste leachate samples were collected on 24 September 2020 at the Olusosun (N 6°29′21.8″; E 003°23′29.3″) and Oke-Afa dumpsites (N 6°27′11.0002″; E 3°23′44.9999″), along with soil samples from the empty lands surrounding Olusosun (6° 35′ 55.25″N; 3° 22′12.09″ E) and Oke-Afa dumpsites (6° 31′ 40.42″N; 3° 19′ 6.46″E).

Genomic DNA was obtained with the use of the ZR fungal/bacterial DNA Kit (Zymo Research, Irvine, CA, USA), following the manufacturer’s instructions. The genomic DNA samples were amplified using a universal primer pair (515F [7] GTGYCAGCMGCCGCGGTAA; 806R [8] GGACTACNVGGGTWTCTAAT) targeting the V4 region of the 16S rRNA. PCR program was run as follows: initial denaturation at 95°C for 3 min, followed by 30 cycles of denaturation at 95°C for 30 s, annealing at 56°C for 30 s, elongation at 72°C for 1 min, and a final elongation step at 72°C for 5 min.

With the use of a MiSeq v3 (600 cycle) kit, the amplicons were sequenced on Illumina’s MiSeq platform, and about 20 Mb of data (2 × 300 bp long paired-end reads) was generated for each sample. FastQC v0.11.9 (9) was used for the quality assessment of the raw amplicon reads. Pre-processing included the use of Trimmomatics v0.39 (10) for the removal of adaptor sequences, low quality (using phred33), and short reads (<100 bp) for both the paired-end files. For sequence denoising methods, filtered reads were further processed using qiime2 v2020.6.0 (11). The denoising method was implemented using DADA2 (qiime2 plugin: q2-DADA2 [12]), with truncating lengths of 284 and 118 for the forward and the reversed reads, respectively. Operational taxonomic units (OTU) were based on amplicon sequence variants (ASV) defined by DADA2. Table 1 shows the solid waste leachate amplicon collection features. Oke-Afa dumpsite had higher reads of 81,940 than Olusosun dumpsite with 68,339 reads. The Olusosun and Oke-Afa dumpsites yielded 305 and 306 OTUs, while the Olusosun control sample yielded 233 OTUs. The Oke-Afa control was excluded due to the low read counts. The Olusosun control provided valuable comparative insights into the impact of waste on the two dumpsites, which exhibit similar characteristics.

**TABLE 1:** Solid waste leachate (SWL) amplicon collection features of Olusosun and Oke-Afa dumpsites

Parameter	Value		
	Olusosun dumpsite	Oke-Afa dumpsite	Olusosun control^*a*^
No. of reads	68,339	81,940	2,429
OTU total count	305	306	233
Inverse Simpson index			
Shannon’s evenness average	1.67	0.851	4.58
Good coverage	99.27	99.25	90.71
Simpson	0.60	0.39	0.97

^*a*^ Empty land around the Olusosun dumpsite.

**Fig 1:**
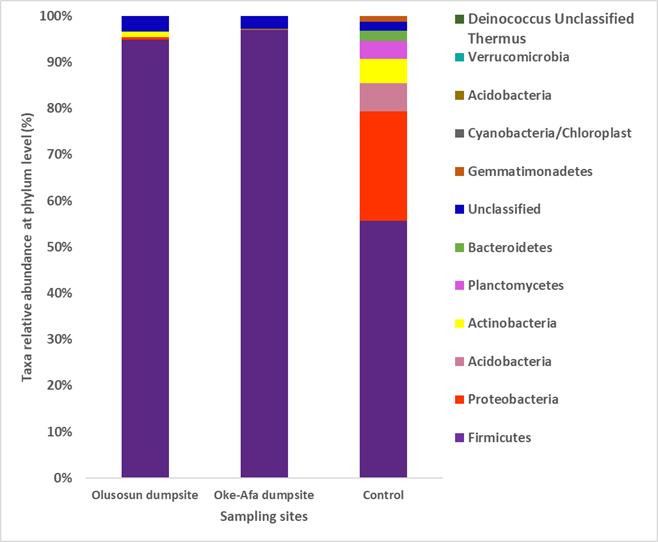
Taxon relative abundance at phylum level.

Figure 1 depicts the taxon relative abundance at phylum level for Olusosun and Oke-Afa dumpsites. The taxon phylum *Firmicutes* had total reads of 94.89 and 96.90 % at the Olusosun and Oke-Afa dumpsites, respectively, with equivalent total reads of 54.18% at the Olusosun control. *Acidobacteria* and *Actinobacteria* had the same lowest total readings (0.07 %) at the Olusosun and Oke-Afa dumpsites.

## Data Availability

The sequence data discussed in this study are available at the NCBI under accession numbers ranging from PV357926 to PV357968. The corresponding SRA accession numbers obtained were SRX29075654 for Oke-Afa dumpsite solid waste leachates (SWL), SRX29075655 for Oke-Afa soil (empty land outside Oke-Afa dumpsite), SRX29075652 for Olusosun dumpsite solid waste leachates (SWL), and SRX29075653 for Olusosun soil (empty land outside Olusosun dumpsite). The BioProject accession for the amplicon sequencing study was PRJNA1269070.
